# From Simple Receptors to Complex Multimodal Percepts: A First Global Picture on the Mechanisms Involved in Perceptual Binding

**DOI:** 10.3389/fpsyg.2012.00259

**Published:** 2012-07-23

**Authors:** Rosemarie Velik

**Affiliations:** ^1^Carinthian Tech ResearchVillach, Austria

**Keywords:** perception, binding problem, combination coding, temporal coding, population coding, focus of attention, knowledge, inhibition

## Abstract

The binding problem in perception is concerned with answering the question how information from millions of sensory receptors, processed by millions of neurons working in parallel, can be merged into a unified percept. Binding in perception reaches from the lowest levels of feature binding up to the levels of multimodal binding of information coming from the different sensor modalities and also from other functional systems. The last 40 years of research have shown that the binding problem cannot be solved easily. Today, it is considered as one of the key questions to brain understanding. To date, various solutions have been suggested to the binding problem including: (1) combination coding, (2) binding by synchrony, (3) population coding, (4) binding by attention, (5) binding by knowledge, expectation, and memory, (6) hardwired vs. on-demand binding, (7) bundling and binding of features, (8) the feature-integration theory of attention, and (9) synchronization through top-down processes. Each of those hypotheses addresses important aspects of binding. However, each of them also suffers from certain weak points and can never give a complete explanation. This article gives a brief overview of the so far suggested solutions of perceptual binding and then shows that those are actually not mutually exclusive but can complement each other. A computationally verified model is presented which shows that, most likely, the different described mechanisms of binding act (1) at different hierarchical levels and (2) in different stages of “perceptual knowledge acquisition.” The model furthermore considers and explains a number of inhibitory “filter mechanisms” that suppress the activation of inappropriate or currently irrelevant information.

## Introduction

The binding problem in perception is concerned with answering the question how information from millions of sensory receptors, processed by millions of neurons working in parallel, can be merged in to a unified percept. Finding an answer to this question is on the one hand crucial for understanding the functioning of the brain and therefore tackles researchers from various disciplines of brain sciences. On the other hand, gaining insight into this topic is also highly valuable for a subfield of engineering called “Brain-Like Machine Perception” (Velik, 2008). Brain-Like Machine Perception is concerned with developing brain-inspired concepts and technologies for a new generation of information processing and automation systems. The motivation for the research presented here comes exactly from this latter discipline and originated from the following challenge: sensor technology is getting smaller and smaller while at the same time becoming cheaper and cheaper. The consequence is that in future, systems like robots or building automation systems will be equipped with a larger number (up to millions) of individual sensors. This will enable completely new application domains. However, today’s technical approaches cannot cope with the processing and interpretation of such a flood of incoming data. Novel concepts are needed (Velik et al., 2011). One way to find a potential solution to this challenge is to take inspiration from the brain – a system that is capable of processing information from millions of sensory receptors and merging them into unified percepts.

Driven by this idea, we formed an interdisciplinary research team of engineers and brain scientists and worked on the development of a technically implementable model of the human perceptual system of the brain – a task which included also an extensive study of the binding problem in perception. During the course of this research, we did not only develop innovative concepts and methods for future engineering systems, but also gained new insights and formulated new hypotheses concerning brain functioning. While engineering aspects of this work and a first draft of the overall model from the viewpoint of cognitive sciences have already been presented elsewhere (see for instance Velik, 2010a,b,c; Velik and Boley, 2010), the article of this special issue shall now particularly focus on a description of newly gained insights and hypotheses concerning the binding and inhibition mechanisms involved in perception. For this purpose, we first give a summary of so far suggested potential solutions to the binding problem and their strengths and weak points in Chapter 2 followed by a presentation of our proposed perceptual binding model in Chapter 3. The model is based on a conclusive combination of so far suggested potential solutions to the binding problem and further supplementary considerations and hypotheses including several inhibition mechanisms coming from feedback connections and top-down guided mechanisms. The principal functionality of the resulting model is validated via computational simulations. The next step to take, which is not covered by this article, is to search for physiological support of the suggested hypotheses by experiments and observations in animals or humans. With this article, we would like to encourage other research groups to join this verification process.

## Strengths and Weak Points of Current Binding Hypotheses

### What is the binding problem? – a first simplified explanation

**Figure 1:**
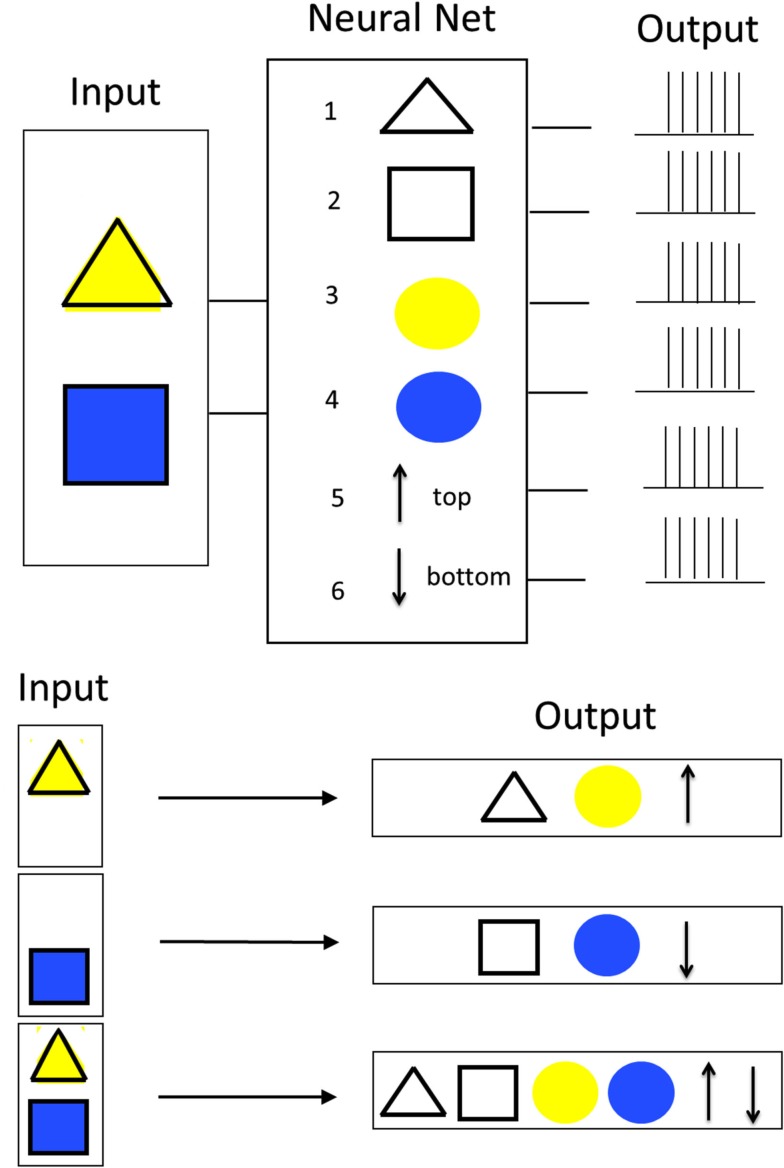
**Classical illustration of the binding problem**.

The binding problem in perception takes its origin in the field of Gestalt psychology, which was concerned with trying to understand by what principles visual features tend to be grouped to particular perceived objects. According the identified Gestalt principles, such a grouping is done based on properties like proximity, continuity, simplicity, closure, similarity, figure-ground distinction, and common fate (movement into same direction). The binding problem as considered today goes a step further and tries to investigate what processing mechanisms lie behind such “grouping effects” within and across modalities. The principal problem that current brain science has with understanding how information is “bound” in perception is probably best explained by a concrete example (see Figure 1). The most extensive discussion of binding has so far concerned binding in visual perception. The presented example constitutes an extended version of F. Rosenblatt’s classical illustration of the binding problem from his book “Principles of Neurodynamics: Perceptrons and the Theory of Brain Mechanisms” (von der Malsburg, 1995, 1999). The example concerns binding of visual information. At the input of a hypothetical neural network consisting of six neurons, different visual images are presented that can either be yellow or blue triangles or rectangles in an upper or lower position. In order to process the incoming information, in analogy to observations made in the visual cortex, different neurons of the network respond to different features of those objects. In the example, two neurons respond to the shape of objects (neuron 1 to triangles, neuron 2 to squares) and two further neurons respond to the color of objects (neuron 3 to yellow, neuron 3 to blue). Last but not least, the two remaining neurons represent the position of the objects (neuron 5 means upper position, neuron 6 means lower position). If now for example either a yellow triangle in the top position or a blue square in the bottom position is presented, always the three corresponding “feature neurons” are activated. However, a problem occurs in the case that not only one but two objects are presented to the network simultaneously. In this case, all six feature neurons are activated concurrently and without further measures, it cannot be concluded which feature belongs to what object. Finding out how the brain solves this issue to come to unified correct percepts is the so-called binding problem. The binding problem is not limited to perception. Similar mechanisms are also necessary for other brain functions including sensor-motor function, memory, and consciousness (Roskies, 1999). For this reason, the binding problem is considered as one of the key questions to brain understanding (Triesch and von der Malsburg, 1996).

Within the last decades, researchers have intensively worked on finding a solution to the binding problem. We already presented an extensive overview and discussion of so far suggested solutions to the binding problem in (Velik, 2010d). In the following sections, we give a summary of this information as far as it is relevant for understanding the model and hypotheses that will be described in Chapter 3. While Section “Proposed Binding Mechanisms” describes individual so far suggested mechanisms, Section “Proposed Combinations of Binding Mechanisms” is concerned with illustrating what combinations of those individual mechanisms have so far been proposed.

### Proposed binding mechanisms

#### Combination coding

In the 1960s, the Noble price winners Hubel and Wiesel (1962) reported that the visual cortex shows a hierarchical organization. Each hierarchical level receives and processes information from earlier levels. This way, incoming information is merged and condensed from layer to layer leading to more and more complex perceptual representations in higher levels that cover information from larger receptive field sizes.

**Figure 2:**
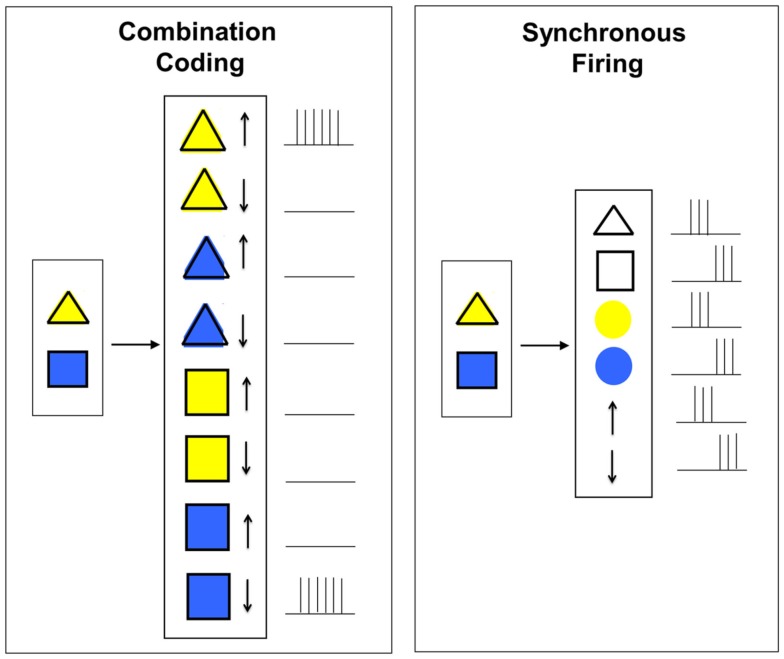
**Combination coding vs. binding by synchronous firing**.

Based on this notion, the hypothesis of combination coding (also called convergent hierarchical coding or grandmother-cell hypothesis) was introduced. Combination coding cells (also called connector cells, cardinal cells, or grandmother cells) always receive convergent input from neurons or populations of neurons of earlier levels and therefore only react to particular combinations of features. This way, incoming information from earlier processing stages is integrated and condensed more and more at higher levels. A simple example for how combination coding can work is given in Figure 2 (left). Similar like in the example in Figure 1, the task that shall be performed by the hypothetical neural network is to detect the simultaneous presence of a yellow triangle in the upper position and a blue square in the lower position. According to the combination coding hypothesis, in order to achieve this task, there has to exist a particular neuron for each possible combination of the features (shape, color, and position). The image representations currently present then result in an activation of the appropriate neurons.

According to combination coding, neural representations are becoming increasingly complex from level to level. At each particular level, a single neuron or a small group of neurons receives convergent input from neurons or populations of neurons from lower hierarchical levels. As noted by Sir Charles Sherrington in his book “Man on His Nature” (1941), following this integration scheme, this could in the extreme case lead to one ultimate pontifical cell as the climax of the whole system. Sherrington however rejected this idea as improbable. In accordance with this, Barlow (1972) suggested that the concept of the pontifical cell should be replaced by a number of “combination cells” from which only a few fire concurrently in order to represent the current perception of the body and the environment.

Evidence for the combination coding hypothesis has been reported particularly for the visual cortex which shows a gradual decrease of retinotopic specificity from layer to layer together with an increase in receptive field size and an increase in complexity of stimulus features to which neurons respond (Hubel and Wiesel, 1962). Models that map these observations of a hierarchical feed forward architecture for simple form recognition have been presented by Riesenhuber and Poggio (1999, 2002).

Although at first sight, the combination coding hypothesis seems to be very intuitive, it suffers from certain weak points. The first criticism concerns the question of how cell connectivity patters of such high specificity should be formed. To acquire them by learning would need many examples of all possible objects, shapes, colors, sizes, rotations at all possible locations of the perceptual field. On the other hand, prewiring cell connections would require the storage of all this information in the genes, which is also unlikely (Triesch and von der Malsburg, 1996). A second criticism concerns the fact that combination coding could need as many connector cells as there are distinguishable objects. If a connector cell was to represent a whole class of objects, it would not be able to represent fine object details. On the other hand, if there existed a cell for all objects showing general similarities but differences in details (e.g., one and the same face with different facial expressions), this would quickly lead to a *combinatorial explosion*. Furthermore, this would mean that many cells would have to be silent for long times (up to decades) before their patterns appear again (von der Malsburg, 1981).

#### Population coding

**Figure 3:**
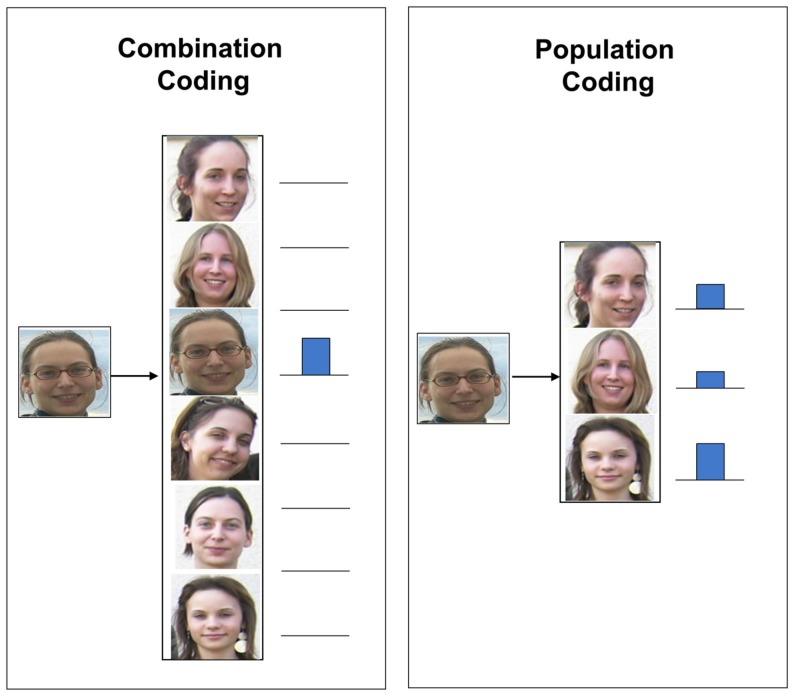
**Combination coding vs. population coding**.

A proposed alternative to overcome the combinatorial explosion of convergent hierarchical coding is population coding (also called distributed coding; Gray, 1999; Goldstein, 2002). The principle of population coding is explained by the example given in Figure 3.

In this example, a particular face shall be recognized. While in combination coding, there would exist a particular neuron (or group of neurons) for each known face, population coding would follows another principle. According to population coding, such complex features are not represented by individual nerve cells but by a whole population of distributed neurons of the same or different cortical levels. A particular sensory input pattern then activates a particular firing pattern in this population. To make this concept plausible, one could imagine that the population of neurons consists of individual neurons representing archetypical faces. If now a particular face shall be recognized, the “archetype faces” are activated more or less strongly depending on the grade of similarity with the presented image thus leading to a specific activation pattern inside the neural population. In comparison to combination coding, by this coding scheme, the representational capacity of the network would be greatly increased as the number of possible distinct stimulus patterns is by far higher than the number of neurons necessary to represent them. Thus the issue of combinatorial explosion would no longer pose a problem.

The theory is supported by physiological evidence from sensory and non-sensory cortical areas and has shown to mesh well with various aspects of the physiological and anatomical organization of the visual cortex. For instance, Haxby et al. (2001) showed using functional magnetic resonance imaging that in the ventral temporal cortex, the representation of faces and objects is widely distributed and overlapping. Similarly, O’Toole et al. (2005) reported for the same brain area that object categories with shared image-based attributes have shared neural structures. Quian Quiroga et al. (2007a,b) conducted experiments in the medial temporal lobe to investigate population vs. combination coding and pleaded for this brain area in favor of a spares but not “grandmother-cell” coding.

Although population coding seems to play an important role in binding, the theory again seems to be incomplete. A problem with this hypothesis arises when more than one object of the same group appears in the same scene. The unanswered question – referred to as *superposition problem* – is how a particular pattern can be identified from the many other patterns represented by the same network concurrently without interference.

#### Synchronous firing

To avoid the combinatorial explosion that would follow from combination coding and furthermore overcome the superposition problem of population coding, the hypothesis of binding by synchronous firing (also called binding by synchrony, temporal binding, or temporal correlation hypothesis) was suggested by Legendy (1970), Milner (1974), and von der Malsburg (1981) who formulated it independently from each other (von der Malsburg, 1999). The basic principle of binding by synchronous firing is illustrated in Figure 2 (right) and suggests that binding can be solved by temporal correlations in firing patterns. The task to perform in Figure 2 is again the same as described in the examples in the Sections “What is the Binding Problem? – A First Simplified Explanation” and “Combination Coding.” With the hypothesis of temporal coding, a temporal dimension is invoked to cell responses. This means that neurons representing features (in our case shape, color, and position) belonging to the same object are correlated in time while neurons representing features of different objects are anti-correlated in time, i.e., their firing patterns are independent. This way, multiple feature combinations can coexist in the same network (Treisman, 1996; Ghose and Maunsell, 1999; Fries, 2005).

The temporal binding hypothesis seems plausible as the output patterns of neurons depend on the precise timing of their synaptic inputs. Ghose and Maunsell (1999) have reported that humans are sensitive to timing differences down to 150 μs. In the last 15 years, experimental evidence from the visual system has been provided that supports the temporal coding hypothesis (Ghose and Maunsell, 1999; Jacobs et al., 2007). Gray et al. (1989) found in-phase neural oscillations for neurons with overlapping receptive fields and non-in-phase oscillations for cells with no such overlap. Amongst others, Gray (1999), von der Malsburg (1999), and Gabriel (2004) suggested a time range of 1–10 ms for synchronous firing. Usrey et al. (2000) support this statement by physiological evidence and report that the time window for reinforcement of heterosynaptic interaction is shorter than 7 ms in the cat geniculocortical pathway. Singer (2001) describes oscillatory modulations of neural firing patterns in the gamma-frequency range (30–90 Hz). Fries et al. (2001) discovered that gamma-frequency synchronization causes spikes to coincide in a range of 10 ms.

Despite these results, the role of synchronous neuron firing in feature binding is still controversial. Grossberg and Grunewald (1996) suggest that synchronization rather has the function of “perceptual framing” of non-constant retinal images. According to them, in case of motion in the retinal image, a mechanism is needed to ensure that parts in the image belonging together are still processed together. Otherwise, illusory conjunctions can occur. Via perceptual framing, parts of an image are re-bound by resynchronizing network inputs with a temporal offset less than a critical delay.

Sharkey (1998) expresses her doubts about temporal binding and points out that there is no evidence that neurons can respond to synchronous input with the precision that would be necessary. Furthermore it is criticized that the observations of synchrony were made in anesthetized animals. The correlation might therefore have been a consequence of anesthesia. Stryker (1989) pointed out that further work is needed demonstrating that those oscillatory phenomena are also definitely present in awake animals. Schultz et al. (2001) analyze the limits in terms of amount of information that can be extracted from an observer from such a synchronous code in order to determine if this amount is sufficient to allow for computational processes like feature binding. However, no final conclusion is drawn by them. Another point of discussion is that observed synchronous firing could also just be an artifact of binding instead of the crucial binding mechanism (Ghose and Maunsell, 1999). The hypothesis of temporal binding is only about how binding is signaled, not about how it is computed. Thus synchrony could be rather the result than the cause for binding. Ghose and Maunsell (1999) point out that this therefore begs the question how synchrony is achieved. Stryker (1989) indicates that the observation of rhythmic oscillations and their correlation with particular stimuli does not allow the conclusion that the brain makes use of this information.

Golledge et al. (1996) indicate that physiologically, the establishment of synchrony is too slow to account for normal object recognition in familiar situations. In order to provide a binding mechanism, synchronous firing would have to occur very close to stimulus onset. However, observations have shown that synchronization is not phase-locked to the onset of the stimulus and starts at a variable time after presentation of the stimulus. Later studies of Fries et al. (2001) and Stefanics et al. (2005) however partly invalidated this criticism by reporting about findings of partly phase-locked stimuli onsets in the gamma-frequency range.

Sharkey (1998) reports that maybe binding by synchronous firing is not computed in the primary cortex but instead imposed via top-down feedback connections from higher levels (see also Top-Down Synchronization).

Ghose and Maunsell (1999) indicate that binding by synchrony was suggested in order to overcome the hypothetical combinatorial explosion emerging with convergent hierarchical coding. They however provide calculations concerning the number of neurons needed in the visual cortex in order to represent all possibly occurring distinguishable visual perceptions and conclude that the combinatorial explosion is not really a problem and that thus temporal correlation is no compelling need for binding. Based on their findings, Ghose and Maunsell (1999) suggest that, although there is no absolute need for temporal binding in everyday life situations, it could be important during recognition learning (see On-Demand vs. Hardwired Coding).

#### Attention

A problem with the hypothesis of binding by synchrony is that spatial information of combined features is lost. Treisman and Gelade (1980) suggested that focused attention plays a crucial role in solving the binding problem. They suggest that visual information is first coded in several separate dimensions including color, brightness, orientation, and direction of movement. Via focused attention, those features are then related to each other to form objects. For this purpose, not all information is processed and integrated simultaneously but is limited top-down to one object per space and time. Once this information is processed, the features of the next object are merged. Thus, information processing is “serialized” via focal attention. The favorite metaphor for visual attention is therefore a spotlight. Only information that is currently in this “mental beam of light” is processed. This way, spatial information of features is obtained indirectly by restricting the current binding area to a certain region (Hommel and Milliken, 2007). This “beam of attention” can either be directed to small areas to obtain information with high resolution or to larger areas which results in a perception with less detail. Chun and Wolfe (2001) suggest that via attention currently relevant information is selected and interfering or irrelevant information is ignored according to the goals and state of the perceiver.

Ghose and Maunsell (1999) indicate that attention may also play a role in differentiating objects which at first glance seem the same. They describe further discrimination as a sequential process that requires sequential processing. This way, perceptions of higher detail level can be achieved.

Treisman and Gelade (1980) report that attention is needed for the correct perception of conjunctions. However, the mechanism of focused attention, by which information processing is “serialized,” cannot be reconciled with the speed with which object recognition can take place. Riesenhuber and Poggio (1999) and Gray (1999) therefore suggest that object recognition does not base on focus of attention alone but that there have to act mechanisms prior to attention, which additionally serve to attract it (see Bundling and Binding).

#### Knowledge, memory, and expectation

While more classic approaches consider the perceptual system and the brain as a whole rather as a passive, stimulus-driven system, more recent theories point out the active nature of perception, which seems to be controlled by top-down processes. Apart from focus of attention, further top-down processes acting on perception and taking a role in binding are semantic knowledge, context knowledge, memory, expectation, and mechanisms related to these concepts. This notion is today supported by various researchers. Engel et al. (2001) indicate that sensory-motor processes, cognition, and behavior are to a large extend not reflex-like, based on incoming (sensory) stimuli, but also heavily influenced by expectations derived from generalized knowledge and experience coded in cortical networks. Similarly, Ernst and Buelthoff (2004) report that for interpreting (ambiguous) sensor signals, prior knowledge is often necessary. Wolfe and Cave (1999) point out that different patterns could be produced by the same stimuli due to different expectations of a subject. Engel et al. (2001) point out that top-down processes assure perceptual and cognitive processing to be fast and reliable. Using these mechanisms, predictions about forthcoming stimuli can be made, which are then continuously matched against signals from the environment and body. Treisman and Gelade (1980), who suggested focused attention to be a binding mechanism, furthermore indicated that contextual information and past experiences take a role in the binding process. Accordingly, it is for instance unlikely that we will perceive a blue sun and a yellow sky even if attention is directed elsewhere. Additionally, they point out that features, once correctly bound by focused attention, continue to be perceived and stored this way. The same observation was made by Hommel (1998) who indicates that not all phenomena can be explained by temporal integration trough attention alone, like for example the experience of object constancy despite changes in some features over time. Without additional mechanisms, as soon as attention is shifted, this information would be lost again. There is thus some kind of memory and knowledge needed acting top-down to preserve the information (Wolfe and Cave, 1999; Engel et al., 2001). In the process of applying top-down mechanisms on incoming stimuli, the prefrontal and parietal cortex seem to play a particularly important role (Frith and Dolan, 1997; Miller and Cohen, 2001).

Models incorporating top-down processes usually suggest that predictions about features of the surrounding are expressed by firing patterns traveling from higher to lower hierarchical processing levels via feedback connections where then a comparison of predicted perceptions with sensory input takes place (Engel et al., 2001). Ullman (1995) indicates that the interaction between top-down and bottom-up processes can occur at any intermediate level. The only condition is that they have to arrive simultaneously. Engel et al. (2001) suggest that a mismatch of bottom-up and top-down signals causes an extinction of signals in early levels while a match leads to an amplification.

Despite its many advantages, the usage of knowledge, memory, and expectation can also be cause of misperceptions. In familiar situations, top-down prediction of objects and events can lead to fast and efficient information processing. However, in unfamiliar situations with misleading context, predictive expectations can lead to wrong interpretations of stimuli. While top-down processes usually interact with incoming sensory stimuli to “create” perception, in some cases, they can even act in complete absence of incoming stimuli. This situation occurs in perceptual hallucinations of subjects with schizophrenia but also in normal subjects during mental imagery (Frith and Dolan, 1997).

### Proposed combinations of binding mechanisms

As reported in (Treisman, 1996) different binding mechanisms suggested so far are not mutually exclusive. Accordingly, as described in the following sub-sections, different authors have already suggested a combination of particular binding mechanisms for explaining feature binding.

#### On-demand vs. hardwired coding

In Section “Synchronous Firing,” it was outlined that temporal coding is particularly flexible and economic in terms of cognitive structure. However, it has been criticized as this mechanism would not be able to perform binding with the speed necessary in familiar environments as even features that are very likely to occur together would need to be bound anew every time. Therefore, Colzato et al. (2006), Hommel and Colzato (2009), and VanRullen (2009) suggested that there exist two distinct binding mechanisms in perception. For frequently encountered and important objects, hardwired binding (combination coding) is applied. For more arbitrary or meaningless feature combinations, an on-demand temporal coding mechanism is used. VanRullen (2009) suggested that on-demand binding is always mediated by attention. In contrast, hardwired binding can work without attention for single objects but needs attention if multiple objects are present in the receptive field.

Golledge et al. (1996) propose that, rather than for the perception of familiar objects, temporal binding might take a particular role in recognition learning, which can have a longer time course. von der Malsburg (1995) indicates that binding by synchrony has a limited bandwidth of neural signals. However, stereotypical tasks show very short reaction times that cannot be explained by temporal binding. Thus he suggests that the more time-expensive synchronous binding is only used for novel situations. Once a cellular binding structure has turned out to have a long term value, it is stabilized into faster but less flexible specialized circuits. This hypothesis would be in line with Hebb’s cell assembly theory (Hebb, 1949) according to which cells that fire together (i.e., show synchronous firing patters) start wiring together until they result in faster and therefore more efficient hardwired structures. Singer (2001) supports this hypothesis by indicating that neural connection achieved via temporal binding can be stabilized through learning. In this process, synchrony could be invoked either via focus of attention or maybe in-phase firing patters of topographically correlated cells.

#### Bundling and binding

In Section “Attention,” attention was suggested as a binding mechanism. However, as described there, attention alone cannot explain the speed with which perception takes place. Accordingly, Treisman and Gelade (1980) proposed for visual perception that conjunctions are also formed in the absence of focused attention, however rather on a random basis. Thus, if attention is overloaded or directed elsewhere, illusory conjunction can occur. In accordance with this view, patients with parietal lobes damage in the regions involved in allocation of attention have shown to lead to illusory conjunctions (Reynolds and Desimone, 1999). Reynolds and Desimone (1999) report that the number of incorrectly bound feature conjunctions increase exponentially with the number of objects in the receptive field. Thus higher receptive levels representing lager receptive fields are more sensitive to erroneous feature conjunctions. Attention is the mechanism to resolve these incorrect feature bindings by restricting the spatial area in which information is processed at a certain moment.

In relation to this, Wolfe and Cave (1999) suggest that binding involves an early pre-attentive and a later attentive stage of processing. In the early levels of processing, i.e., the primary visual cortex, features are represented by spatially organized (topographic) maps of the visual field. This means that neighboring neurons in the retina project their information on neighboring neurons in the primary cortex. Thus, at those lowest levels, this implicit location information serves as means for interrelating features belonging to a particular object and therefore prevents features from “free floating.” Nevertheless, although all necessary information is present at those early levels, those features are rather “*loosely bundled together than tightly bound*.” Without attention, it is probably not possible to recode those interrelations of features into memory. In higher processing levels, the specific location information of features is no longer available as there, information is no longer arranged topographically. Therefore, selective attention is necessary for binding it at those higher stages.

#### Feature-integration theory of attention

According to the feature-integration theory of attention suggest by Treisman and Gelade (1980), which shows some similarities to the bundling and binding theory, features like color, brightness, direction of movement, orientation, etc. are detected automatically and in parallel in early levels of visual processing. In contrast, objects are registered separately in later stages. Awareness for objects is obtained in two different ways: (1) through focused attention and (2) via top-down processes basing on contextual knowledge and memory of past events/percepts. Usually, those processes work synergistically. However, in extreme cases, they might work almost independently.

#### Top-down synchronization

In Section “Synchronous Firing,” it was outlined that synchronous neural firing might play a role in binding. However, the question what the function of neural synchrony in early processing levels is and how it can be achieved is controversial. Several authors suggest that synchronous neural firing might be imposed via top-down feedback connections from higher cortical areas representing functional relationships during stages of attention or expectancy (Sharkey, 1998; Engel et al., 2001; Fries et al., 2001; Fries, 2005). In the process of synchronous firing, also different neurotransmitter systems (e.g., cholinergic or dopaminergic) and astrocytes could theoretically play a role, which are however not further discussed in the current article.

Fries (2005) proposes that via the top-down mechanisms, a modulatory input is provided to selected neural cell groups of earlier cortical levels. In the case of attention, neural groups are defined by their topographic position in the receptive sensor map. Thus, by synchronous firing induced by attention mechanisms, currently relevant areas are selected and transformed from a spatial (topographic) code to a temporal code. Apart from locations, attention can also be directed toward different modalities or particular object features (e.g., color or movement; Engel et al., 2001). In support of this hypothesis, recent studies of Fries (2005) have shown that spikes coming from neurons representing attended features are more precisely gamma-band synchronized than the spikes of neurons representing unattended stimuli. Fries (2005) further reports that thalamic nuclei and other “broadcasting centers” with widespread reciprocal connections within the cortex could take over the function of distributing the synchronization rhythms. Selectivity could be achieved via specific rhythms sent to particular areas. Engel et al. (2001) report about studies according to which, apart from attention, also states of “anticipation” and “expectancy” can be represented via temporally synchronized activity patterns that occur before the appearance of stimuli.

Engel et al. (2001) suggest that the synchronization effect caused by attention is detectable in the primary cortical areas. Nevertheless, the extent to which synchronization can be observed increases in higher cortical levels. However, this effect might not be caused by attentional mechanisms (alone) but also by knowledge, memory, and expectation.

## Integrative Solution

In Chapter 2, different so far suggested hypotheses concerning the binding problem have been presented. Each of the proposed mechanisms seems to address some important aspects of binding. Nevertheless, none of them could so far give a complete answer. As the different solutions are however not contradictory, a conclusive combination of them might lead to a more satisfactory explanation of how binding in perception works. In this Chapter, such an “integrative” solution is proposed and the underlying perceptual model is described.

### Model overview

**Figure 4:**
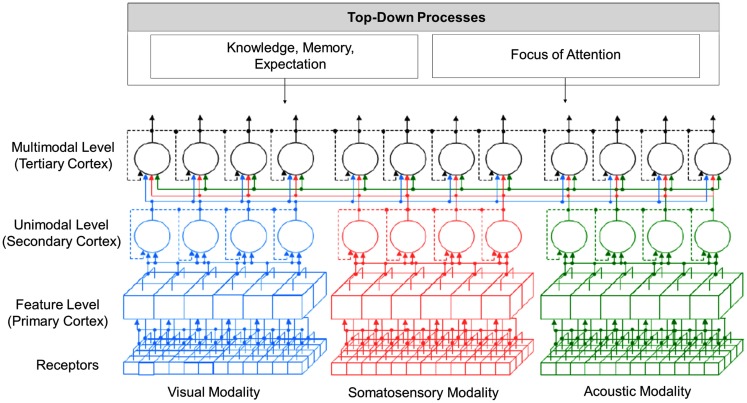
**Overview of “integrative” solution to the binding problem**.

In Figure 4, an overview of the proposed model is presented. The model covers perceptual information processing from the level of sensory receptors up to the level of multimodal perception and includes the visual, auditory, and somatosensory modality. Following research findings reported by Luria (1973), perceptual information processing in the model is divided into three levels, from which each level can consist of several sub-layers (Velik, 2008). In the first two levels, corresponding to the function of the primary and secondary perceptual cortex, information for each sensory modality is mainly processed separately and in parallel (see Representation of Location Information for exceptions). In the first level, neurons – here represented by cubes – respond to relatively simple features. For the visual modality, examples for processed features are lines, edges, colors, or movements in to a certain direction always at a certain location of the receptive field. Examples for the acoustic modality could be sounds of a certain frequency. The primary cortices are topographic, meaning that for each modality, neighboring receptors project on neighboring neurons in the cortex. Information is therefore highly location dependent. On the second level, activated neurons (or groups of neurons) – here represented by circles – respond to whole unified percepts of each modality. Examples for the visual modality would be faces, objects, persons, etc. and for the acoustic modality voices, melodies, etc. Representations are independent of the concrete location, orientation, size, etc. of the perceptual images. On the third level, corresponding to functions of the tertiary cortex, information from different sensory modalities is merged. An example for processing in this level would be the correlation of a visual image of a person with a voice to recognize that those two percepts belong together and that a particular person is currently talking.

The model shows a feedforward “simple-to-complex” hierarchy as reported by Hubel and Wiesel (1962) but additionally comprises feedback connections (see Feedforward and Feedback Connections). Furthermore, perceptual processing does not only depend on information coming from receptors but is also heavily influenced by top-down processes like knowledge, memory, and expectation (see Focus of Attention) and focus of attention (see Representation of Location Information).

### Feedforward and feedback connections

**Figure 5:**
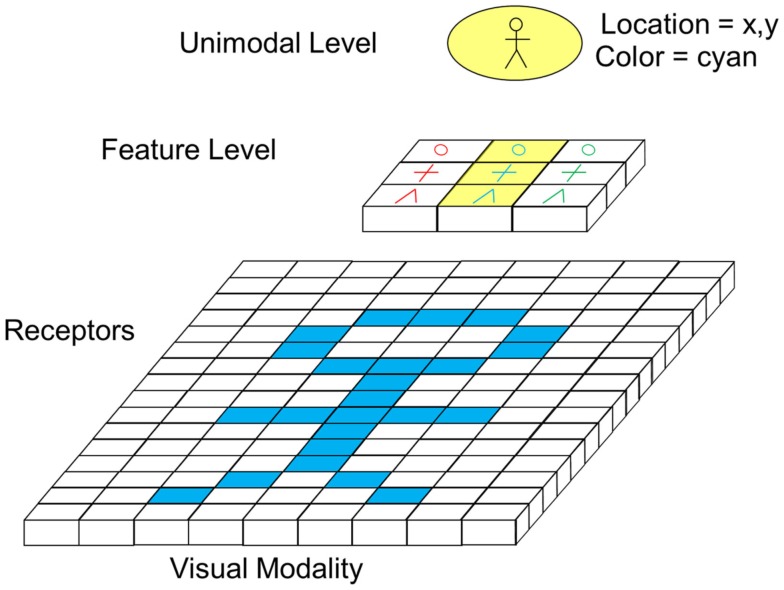
**Feedforward visual information processing from receptor level to unimodal level (excitatory forward connections not depicted)**.

A clarification of the function of the different layers and their interconnections is probably best explained by concrete (simplified) examples. Figure 5 shows an example schematically illustrating visual feed forward information processing from the receptor level up to the unimodal level.

In the example, a “person symbol” in cyan color shall be recognized. For this purpose, receptors are connected to cells of the feature level and cells of the feature level are connected to the unimodal level via exhibitory connections. For reasons of clarity, the connections are not depicted in the figure. Activated cells are highlighted with yellow color. The feature level shows a topographic structure, meaning that neighboring receptors project on neighboring feature cells. As indicated by its name, cells of the feature level respond to different features of a visual image. In the given example, cells respond to shapes like circles, crosses, or edges of a specific color. In Figure 5, just one segment of the whole visual field is shown. Other segments of the visual field project on other cells of the feature layer. Cells on the unimodal level now receive input from feature cells of different sectors. In the concrete example, the depicted cell recognizes a person if a circle, a cross, and an edge feature are recognized in the right spatial arrangement at a certain location. Unlike assumed in F. Rosenblatt’s classical illustration of the binding problem (see Figure 1), this model therefore suggests that location information on the feature level is not just an additional feature like color, shape, and orientation but the crucial mechanism for binding, which is at this level coded in the spatial arrangement of cells. From the unimodal level upwards, spatial integration of information is no longer achieved via topographical representations but coded by other means (see later sections). Options for this could be specific firing patterns, the activation of cross-modal neurons, or focus of attention. At the unimodal level, cells generally respond to visual images (e.g., a person) independent of the color, size, orientation, etc. of the image except if those features are very characteristic for the object. Therefore, like for location information, these characteristics have to be coded by additional means.

**Figure 6:**
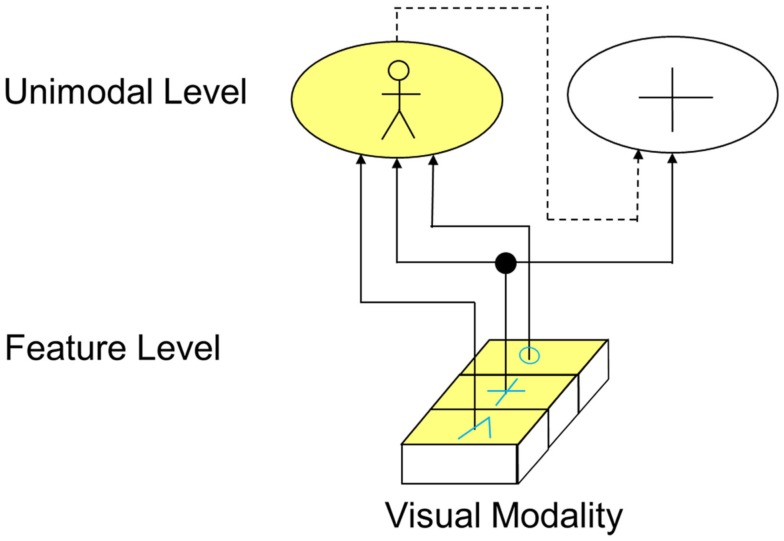
**Function of feedback connections (solid lines indicated excitatory connections, dotted lines indicate inhibitory connections)**.

Besides feedforward connections, the visual cortex also shows a number of feedback connections. The function of those connections is however not yet well understood. In the example of Figure 6, it is shown what important role inhibitory feedback connections can have in perception. Let’s assume that, similar like in the example of Figure 5, three neighboring cells representing a circle, a plus, and an edge are active at the feature level. On the unimodal level, there does however now not only exist a cell representing a person but another cell representing a cross. Via the depicted exhibitory forward connections alone, now both the “person cells” and the “cross cells” would be activated. However, the activation of the “cross cell” would be inappropriate. To avoid this concurrent undesired activation, there exists an inhibitory feedback connection (depicted as dotted line). By this means, the activation of the “cross cell” is deactivated as soon as the “person cell” representing the actual perceptual image is activated.

### Focus of attention

**Figure 7:**
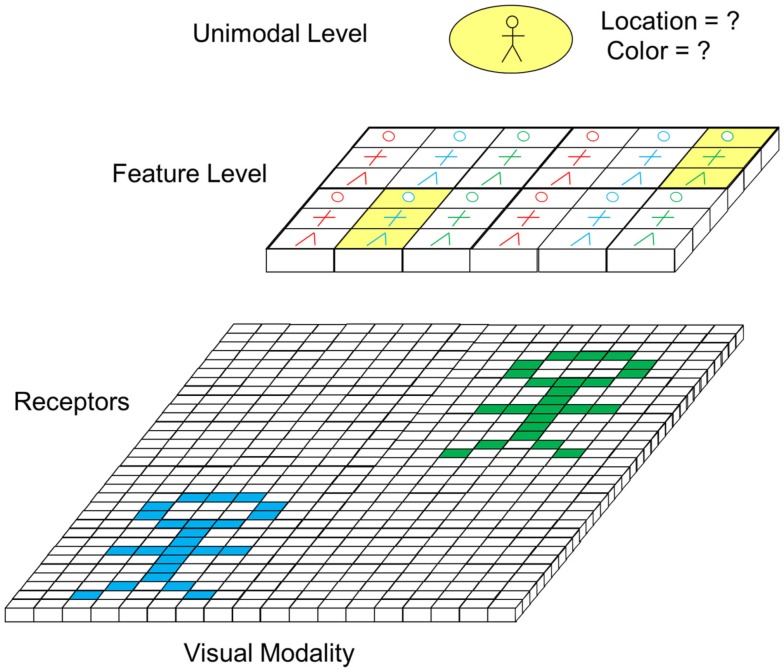
**Potentially ambiguous perception in case of presence of multiple objects (excitatory feedforward connections not depicted)**.

A further mechanism taking an important role in perceptual binding is focus of attention. Focus of attention comes in to play if several objects are present in the environment concurrently. This is well illustrated by the example of Figure 7. There, two person symbols, one in cyan and one in green are present in the visual field at the same time at different locations. On the feature level, which is topographic, this would lead to an activation of feature cells in two different areas (marked in yellow). On the unimodal level, this could lead to an activation of the “person cell.” However, at this level, a binding problem occurs as two persons are present and no conclusive information about the location and color (i.e., the details) of the person symbol can be obtained. To resolve this problem, focus of attention can be applied.

**Figure 8:**
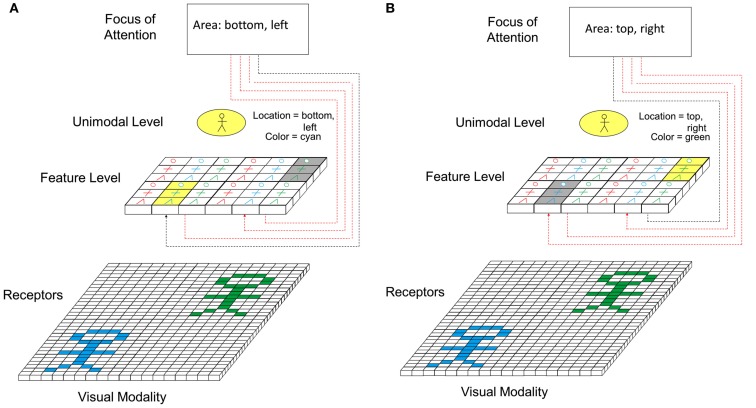
**Role of focus of attention in perception**. **(A)** Focus of attention is switched to lower left part of perceptual field. **(B)** Focus of attention is switched to upper right part of perceptual field.

Figure 8 shows the principle how focus of attention interacts with perception. In the model, focus of attention interacts on the feature level. Via inhibitory connections from the focus of attention, the activation of all feature cells outside a certain spatial range is reduced in comparison to the range to which the focus of attention is currently directed. This way, only information currently “inside” the focus of attention is further processed in higher levels (see Figure 8A). Once processed, the focus of attention is shifted to the next area (see Figure 8B) and the features activated there are now processed. Apart from directing focus of attention toward particular spatial areas of the perceptual field, it is also conceivable that focus of attention can be directed toward particular features (e.g., particular colors, particular shapes, etc.). Seen form a physiological perspective, focus of attention could be represented by a top-down-induced pattern of synchronous firing of neurons representing features being currently in the focus of attention.

### Representation of location information

As already outlined in the previous sections, information about the location where particular features, objects, and the like are perceived has an important role in binding. This becomes particularly important for correctly binding features to higher-level concepts in case multiple objects/events occur at the same time. As explained in Section “Feedforward and Feedback Connections,” up to the feature level, location information about features is represented topographically. Thus binding in those first layers particularly occurs between features represented by spatially proximal neurons. However, the unimodal and multimodal level of perception no longer show these topographic representations. Thus other mechanisms need to act in later levels of perception to code location information. A particularly interesting question in this context is how unimodal representations of different sensory modalities are bound to correct multimodal perceptions if different objects and events occur at the same time. One example could be that two persons are currently perceived in a room at different locations from which one is currently talking and the other is not. How can perceptual information be correctly bound in order to perceive which of the two persons is currently talking? Apparently, the information where in the room a voice was heard and where in the room the two persons were seen has to be matched adequately and the acoustic and visual information having been perceived in an overlapping spatial range has to be merged while the other visual information should not be considered. One possible way to achieve this merging is the mechanism of focus of attention introduced in Section “Focus of Attention.” In this case, the spatial range within which information is considered and therefore can be merged would be limited in each moment of time. The question is however if focus of attention is the only mechanism available as it is quite exact but relatively time consuming. As suggested in Section “Synchronous Firing,” synchronous firing of neurons could have the function to translate the topographic representation of location information of the feature level into a temporal representation in the unimodal level and above. One mechanism to induce synchronous firing could be focus of attention, which however has the disadvantage to be relatively slow. Therefore, other alternatives are conceivable. On the one hand, theoretically, concurrently activated neurons of the feature level in a proximal spatial range could produce such a firing pattern. However, when going beyond information processing for just one particular modality, the question is how synchronization in firing between different sensory modalities can be achieved for representing the same spatial ranges. This would be necessary for multimodal merging of information. One possible mechanism suggested here for achieving this inter-modal synchronization could be cross-modal (i.e., multimodal) cells in levels lower than the multimodal level having the function to spatially interrelate representation of different modalities. Until recently, the general view in neuroscience was that multimodal integration is mainly limited to higher cortical levels after extensive unisensory processing has taken place (i.e., in the multimodal level of our model). However, recent studies report that cells responding to activations of more than one sensor modality can already be found in lower levels of perception (Ghazanfar and Schroeder, 2006; Cappe et al., 2009). The question that has so far not been completely clarified is what the function of those cross-modal neurons is. As outlined above, we propose that their function (or at least one of their functions) is to establish a “spatial correlation” between different modalities. This suggestion is in accordance with findings from Pascual-Leone and Hamilton (2001) who report about recordings of patterns from cross-modal neurons in the visual cortex responding to both visual and acoustic stimulation. Results showed that none of these neurons demonstrated a frequency tuning curve comparable to what can be obtained in the primary auditory cortex. Instead, the acoustic responsiveness of the “audio-visual” cells depended on the location of the sound source in space. Accordingly, they conclude that those neurons are engaged in the representation of the spatial localization of stimuli independent of the sensory modality. Therefore, these cells could play an essential role in “location-sensitive” binding of stimuli of different modalities.

### Knowledge and memory

**Figure 9:**
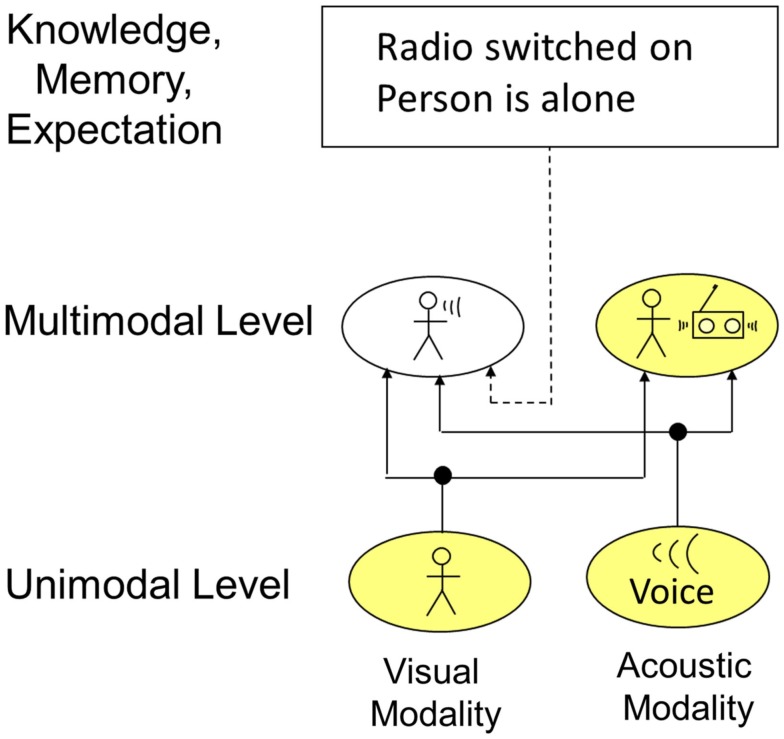
**Influence of knowledge, memory, and expectation on perception**.

In certain situations, perceptual information originating from sensor values can be ambiguous. Furthermore, perception needs mechanisms to preserve the outcome of feature binding to avoid continuous reprocessing of information and to be able to consider former relevant percepts no longer activating sensory receptors. Top-down processes like knowledge, memory, and expectation can help to resolve “conflicts” and store processed information. An example for how the interaction of those top-down processes with perception takes place is given in Figure 9. The example shows information processing on the unimodal and multimodal level. In the visual modality, the presence of a person is detected. In the acoustic modality, a voice is recognized. On the multimodal level, this information could now lead to two different conclusions: (1) the person is talking, (2) the person is listening to the radio positioned right next to him. Taking just the current time instant is not enough to reach an unambiguous recognition. Therefore, memory and knowledge interact at this level. By these mechanisms, it can be memorized that the person switched on the radio several minutes ago. Additionally considering that the person is alone in the room and usually does not talk to himself, it can be concluded that the person is listening to the radio. Accordingly, the activation of the cell representing the “person talking” is deactivated by those top-down mechanisms via inhibitory connections. Principally, the interaction of perception and knowledge, memory, and expectation can take place at every level. However, simulation results showed that interaction at higher levels corresponding to the unimodal and multimodal level are more efficient and therefore more likely. Furthermore, inhibitory as well as exhibitory top-down connections would principally be possible. However, computer simulations and system theory showed that a too large number of excitatory top-down connections can negatively influence system stability (Velik, 2008).

### Overview of involved binding mechanisms

**Figure 10:**
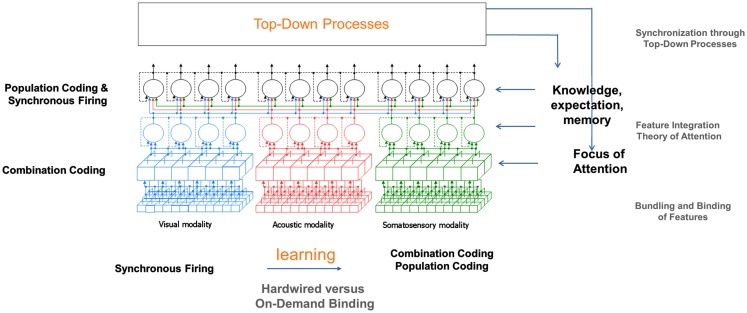
**Overview of binding mechanisms in perception at different hierarchical levels**.

Based on the processing mechanisms described in the last sections, this section shall now give a suggestion what binding mechanisms are applied at different levels of perception (see Figure 10). One central point that has to be considered is that information about the location of perceptual images of one layer is crucial for a correct binding in the next higher layer.

The model suggests that at the feature level, which is topographic in structure, combination coding is the means of choice for binding as at this level, the activation of cells is highly specific to particular features concerning shape, color, movement, etc. at a certain position. Information about the location of features is coded in the topographic maps. The represented features are features that occur very frequently in the environment. Therefore, combination coding is the most efficient option to handle it. The parallel coding assures that all information in the perceptual field is quickly accessible. Only in later stages, filter and selection mechanisms are applied to reduce the amount of information that has to be processed at each time instant.

On the unimodal and multimodal level, a combination of population coding and binding by synchrony seems to be the dominating binding mechanisms. At his levels (groups of), cells are activated by particular perceptual images independent of the concrete location, size, orientation, etc. of those images. Location information is no longer represented topographically but via other mechanisms. A possible candidate for preserving this information is some kind of temporal pattern. Further mechanisms involved could be cross-modal cells responding to features of two or more modalities concurrently and focus of attention (see Representation of Location Information). The transition from the feature level to the unimodal level is of course no abrupt junction but rather a continuous change over the layers from smaller to larger perceptual fields and accordingly from more location specific and simple to less location specific and more complex features.

Additionally to the “bottom-up” processes just described, the mechanisms of focus of attention and knowledge, memory, and expectation support perceptual binding in a top-down manner. A description of how this interaction takes place has already been given in Sections “Focus of Attention” and “Knowledge and Memory.”

Concerning binding in the lower levels of perception, particularly the feature level, it is conceivable that at early development stages, on-demand binding is prevalent basing on binding by synchrony and focus of attention. Only later on, commonly occurring feature combinations become hardwired. To achieve this, Hebb’s law of correlations in activations could come into action: Connections of cells being frequently activated concurrently are strengthened more and more until they represent particular feature combinations in a stable way.

The remaining so far suggested “combined” binding hypotheses (see Feedforward and Feedback Connections) are simply a combination of a subset of the above mentioned mechanisms.

The bundling and bounding theory correlates to the described topographic binding mechanisms at the feature level plus the “focus of attention”-restricted processing in higher levels to allow unambiguous binding. However, unlike the model suggested here, the bundling and bounding hypothesis makes no concrete statement on how and at what level focus of attention interacts with perception. In comparison to the bundling and bounding theory, the feature-integration theory of attention additionally considers the mechanism of knowledge, memory, and expectation. However, again, in contrast to the above described model, no concrete statement is made about the possible ways of interaction. Finally, the theory of top-down synchronization covers aspects of the concepts of binding via focus of attention and binding via knowledge, memory, and expectation. However, once more, prior to this model, no statements about the concrete ways of interaction were made.

## Conclusion

In this article, an overview was given about so far suggested solutions to the binding problem in perception. It was shown that the different existing solutions are not contradictory but that it is actually very likely that all of them play a crucial role in binding, however each of them only at specific hierarchical levels of perception and during specific periods of “perceptual knowledge acquisition.” Accordingly, a new model for perceptual binding was suggested.

To our knowledge, prior studies about binding have mainly focused on the visual cortex and either on a description of one individual binding mechanism only or (to a much less extend) a combination of two (or at maximum three) concepts. However, an integration of the full range of binding mechanisms in to one conclusive model, which reaches additionally from the receptor level up to the multimodal level of perception, has not been provided yet. This article presented a model answering the question what binding mechanisms act at what level in what way and how the interaction of the different mechanisms can take place. We think that having available such a first “global” model will make it much easier to elaborate further details on specific binding mechanisms in different areas and hierarchical levels (e.g., by applying dynamic system theory) and also to integrate newly upcoming insights (e.g., if neurotransmitter systems or astrocytes play a role in binding). Computer simulations (Velik, 2008; Velik and Boley, 2010) showed that this “integrative” concept of binding can provide a conclusive and feasible solution for merging sensory information. The next step is now to validate the model by searching for physiological evidence of the hypotheses presented. This work can however not be performed by one single research group alone. With this article, we would therefore like to encourage the research community to validate our model and hypotheses and to either confirm their validity or to provide constructive critique and/or suggestions for adaptations.

## Conflict of Interest Statement

The author declares that the research was conducted in the absence of any commercial or financial relationships that could be construed as a potential conflict of interest.
